# The Willingness to Pay for Telemedicine Among Patients With Chronic Diseases: Systematic Review

**DOI:** 10.2196/33372

**Published:** 2022-04-13

**Authors:** Valerie Chua, Jin Hean Koh, Choon Huat Gerald Koh, Shilpa Tyagi

**Affiliations:** 1 Office of Healthcare Transformation Ministry of Health Singapore Singapore

**Keywords:** willingness to pay, telemedicine, chronic disease, patients, systematic review, mobile phone

## Abstract

**Background:**

Telemedicine is increasingly being leveraged, as the need for remote access to health care has been driven by the rising chronic disease incidence and the COVID-19 pandemic. It is also important to understand patients’ willingness to pay (WTP) for telemedicine and the factors contributing toward it, as this knowledge may inform health policy planning processes, such as resource allocation or the development of a pricing strategy for telemedicine services. Currently, most of the published literature is focused on cost-effectiveness analysis findings, which guide health care financing from the health system’s perspective. However, there is limited exploration of the WTP from a patient’s perspective, despite it being pertinent to the sustainability of telemedicine interventions.

**Objective:**

To address this gap in research, this study aims to conduct a systematic review to describe the WTP for telemedicine interventions and to identify the factors influencing WTP among patients with chronic diseases in high-income settings.

**Methods:**

We systematically searched 4 databases (PubMed, PsycINFO, Embase, and EconLit). A total of 2 authors were involved in the appraisal. Studies were included if they reported the WTP amounts or identified the factors associated with patients’ WTP, involved patients aged ≥18 years who were diagnosed with chronic diseases, and were from high-income settings.

**Results:**

A total of 11 studies from 7 countries met this study’s inclusion criteria. The proportion of people willing to pay for telemedicine ranged from 19% to 70% across the studies, whereas the values for WTP amounts ranged from US $0.89 to US $821.25. We found a statistically significant correlation of age and distance to a preferred health facility with the WTP for telemedicine. Higher age was associated with a lower WTP, whereas longer travel distance was associated with a higher WTP.

**Conclusions:**

On the basis of our findings, the following are recommendations that may enhance the WTP: exposure to the telemedicine intervention before assessing the WTP, the lowering of telemedicine costs, and the provision of patient education to raise awareness on telemedicine’s benefits and address patients’ concerns. In addition, we recommend that future research be directed at standardizing the reporting of WTP studies with the adoption of a common metric for WTP amounts, which may facilitate the generalization of findings and effect estimates.

## Introduction

### Background

Telemedicine is described by the World Health Organization as “healthcare service delivery by healthcare professionals who use information and communication technologies to exchange valid information for disease prevention, diagnosis, treatment, research and evaluation, as well as for the education of healthcare providers.” This is of interest, considering the need to advance individual- and community-level health [1]. It can be said that the 21st century, despite its challenges, represented a bulk of opportunities for the transformation of health care as well as opportunities for leveraging telemedicine’s potential in enhancing access to care, reducing travel and waiting times of patients, unnecessary emergency department visits, and the misuse of medication [2]. This is made possible by telemedicine as a platform for patient-practitioner contact with a multidisciplinary team, monitoring of vitals, and symptom assessment [3].

The advancement of telemedicine may be attributed to the rising rates of chronic diseases and increasing disease burdens, with chronic diseases such as chronic obstructive pulmonary disease, asthma, and diabetes representing 11.1 million disability-adjusted life years in high-income countries or 7% of the total disability-adjusted life years [4]. Telemedicine has since increasingly been adopted for supporting and integrating care processes in chronic disease management. This encompasses areas such as educating patients to enhance their self-management, enabling the transfer of information from home-based to clinical settings (ie, telemonitoring), and facilitating contact with health care professionals [5]. On March 11, 2020, the World Health Organization declared the COVID-19 pandemic, suggesting the need for prolonged social distancing [6]. The pandemic’s onset has also greatly contributed to the growth of telemedicine use, which enabled distant health care visitations that helped mitigate potential transmissions [7].

While revolutionizing the health care landscape as a new model of care, telemedicine has also been lauded in view of the way it promotes and facilitates self-management [8]. Self-management is defined by the Institute of Medicine as “systematically providing education and supportive interventions by healthcare staff to patients in order to enhance patients’ skills and confidence when it comes to managing their health conditions” [9]. Equipping patients for self-management also includes assessing patient progress and issues, partnering patients for goal setting, and offering problem-solving support [10]. Self-management in itself is deemed crucial because it primarily leads to changes in self-efficacy or the individual’s confidence in managing their conditions. This potentially has positive and direct influences on health status and use [11]. Practical ways in which telemedicine may facilitate self-management are through areas such as enhancing patient-practitioner contact with a multidisciplinary team [3], educating patients, enabling monitoring and feedback provision, allowing for remote clinical reviews, supporting treatment adherence and lifestyle interventions, and intensive interventions [12].

Self-management has also increasingly been integrated into telemedicine-facilitated chronic disease management because patients who have more than one chronic illness require collaborative care and education in the self-management of chronic disease to ensure the best outcomes for patients [13]. The benefits of telemedicine-aided self-management are also apparent from a systematic review conducted by Hanlon et al [14], where telehealth interventions were found to be appropriate and effective in supporting chronic disease self-management. For example, diabetes and heart failure have the greatest evidence base for positive outcomes following telehealth-supported self-management for disease control and health care use. Although evidence on the impact of telemedicine-based self-management for other types of chronic diseases, especially cancers, is limited, overall, there were no adverse effects reported for any other chronic condition assessed in the study [14].

In addition to telemedicine’s efficacy, it is important to also collect information on patients’ willingness to pay (WTP) for telemedicine services, as WTP also serves as a surrogate for the demand and acceptability of such services. WTP is defined as the maximum quantity of resources that buyers are willing to forgo during a transaction in exchange for an object. In considering one’s WTP, the buyer would evaluate if the trade is beneficial for themselves and would subsequently make the purchase when their WTP is greater than the cost of the object sold [15]. As a concept, WTP is increasingly being used to inform health care policy development [16] whereby developing an understanding of WTP is pertinent to aid decision-making. This occurs as knowing the demand for telemedicine services will inform processes such as planning for sustainable financing, resource allocation, or developing a pricing strategy for telemedicine services. It is also important to study the factors influencing patients’ WTP, as this information would be helpful for designing interventions to further enhance WTP. Broadly, WTP may be assessed through the revealed WTP approach and stated preference approach [17]. Specifically for the stated preference approach, the main methods used are

the contingent valuation method (CVM) and discrete choice experiments (DCEs) [18]. CVM involves directly reporting one’s WTP for a particular good through methods such as questionnaires and bidding games. It is the most commonly used method of assessing nonmeasurable economic benefits or costs for goods not present in the market at the point of assessment, thus allowing for the estimation of hypothetical goods’ monetary value [19]. DCEs elicit preferences and are often used to understand the WTP of various attributes or characteristics of the product. This occurs when people are made to select between alternatives featuring the attributes [20].

### Objectives

From our preliminary research, most studies evaluating telemedicine interventions focus on cost-effectiveness analysis [21-24] with limited to no focus on WTP for such interventions. Although cost-effectiveness analysis is important for a health system or policy makers to decide whether to provide financial support for cost-effectiveness interventions, it lacks the patients’ perspective (ie, whether patients will adopt this intervention and would be willing to pay partially or fully if such intervention were to be made available). To address this gap, we aim to conduct a systematic review to describe the WTP for telemedicine interventions, and to identify the factors influencing WTP among patients with chronic diseases in high-income settings.

## Methods

### Information Sources

A systematic literature search was conducted from May to July 2021, abiding by the PRISMA (Preferred Reporting Items for Systematic Reviews and Meta-Analyses) guidelines as presented in Multimedia Appendix 1 [25]. The databases reviewed were PubMed, PsycINFO, Embase, and EconLit.

### Search Strategy

The search strategy was developed on PubMed before translating it to other databases by 1 author (VC) in consultation with the senior author (ST) and university librarian. The main concepts used in this study were *willingness to pay*, *telemedicine*, and *chronic diseases*. For chronic diseases, we included both indexed terms and free-text terms for *chronic diseases*, as well as the 20 chronic conditions included in the Singapore Ministry of Health’s Chronic Disease Management Programme [26]. Although synonyms and variations in spelling for the keywords under each of the main concepts were combined in the search strategy using *OR*, each of the concepts were combined using *AND*. For the search, there were no limits with respect to the publication date, although only studies published in English were included in the systematic review. The detailed search strategy for PubMed is included in Multimedia Appendix 2.

### Eligibility Criteria

Studies were included if they (1) reported the WTP values for telemedicine or identified factors associated with patients’ WTP for telemedicine, (2) included patients aged ≥18 years with chronic diseases, and (3) were based in high-income settings. For our study, high-income settings refer to the countries included under the World Bank list of high-income countries [27]. Only observational studies (cohort, cross-sectional, and case-control studies) and interventional studies were included in the review. Studies were excluded if they were cost-effectiveness analysis studies, protocols, questionnaire validation studies, commentaries, qualitative studies, debates, editorials, newsletters, conference proceedings, letters, and policy reviews.

### Study Selection

A preliminary screening of titles and abstracts to exclude articles that were clearly irrelevant to the eligibility criteria was conducted by 1 author (VC). Abstracts of the remaining articles were screened by 1 author (VC) and verified by a second author (ST) to identify potentially relevant articles. Subsequently, full text was retrieved for selected articles and independently assessed for eligibility by 2 authors (VC and ST). After agreement on the list of articles, the reference list of included articles were screened for additional relevant references.

### Data Extraction and Synthesis for Study Characteristics, WTP for Telemedicine, and Factors Associated With WTP

Data were extracted from the finalized articles into a data extraction sheet. The extracted data included the aim of the study, the study design, the setting, recruitment, eligibility, the total sample size, the telemedicine intervention or service, the type of intervention, the independent variable, the measure of the independent variable, prior experience with the intervention, the outcome, how WTP was measured, the measure of the outcome, whether the data collected were qualitative or quantitative, the data collection method, the analysis, findings, effect estimates, population descriptions, strengths, and limitations.

This paper uses narrative synthesis as its analytical approach. Narrative synthesis descriptively analyzes and draws comparisons across the studies. This approach is appropriate for our systematic review, as the included studies were heterogeneous, limiting the possibility of statistically pooling effect estimates.

### Quality Assessment

The quality assessment for the finalized articles was conducted by 2 authors (VC and JHK) with the help of the Centre for Evidence-based Management guidelines, which may be used to assess the value, relevance, and trustworthiness of cross-sectional studies [28]. Each publication was graded twice independently by VC and JHK. The results in the table were agreed upon by both the authors. There were few disagreements between gradings, and they were resolved in consultation between the authors and a senior coauthor.

## Results

### Study Selection

Our database search yielded 195 results, of which 184 (94.4%) records were excluded after the title and abstract screening, as well as in consideration of our study’s eligibility criteria. A total of 11 articles were included in the final analysis. The study by Losiouk et al [29] was included in our review despite the inclusion criteria stating only to consider patients >18 years with chronic diseases, as the intervention of telemonitoring children with diabetes was meant for parental use.

### Data Extraction and Synthesis for Study Characteristics, WTP for Telemedicine, and Factors Associated With WTP

#### Study Characteristics

The characteristics of the studies included in our systematic review are summarized in Table 1. Of the 11 articles, 3 (27%) were from the United States [30-32], 2 (18%) were from Italy [29,33], 2 (18%) were from Australia [34,35], 1 (9%) was from the United Kingdom [36], 1 (9%) was from South Korea [37], 1 (9%) was from Norway [38], and 1 (9%) was from Belgium [39]. The sample size of the included studies ranged from 23 to 350. The studies’ telemedicine interventions can be largely classified into the diagnosis and management of chronic diseases such as cardiovascular diseases [30,32,33,36], diabetes [29,31,37], and skin cancer [34,35]. For the study by Bergmo and Wangberg [38], it was not specified which chronic disease the telemedicine intervention targeted. To estimate WTP, of the 11 studies, 2 (18%) used the CVM, 4 (36%) used a DCE, 1 (9%) used conjoint analysis, 2 (18%) used surveys, and 2 (18%) used questionnaires.

**Table 1:** Summary of included studies (N=11).

Study	Country	Year	Eligibility	Sample size, n	Intervention	Measurement method for WTP^a^	Patients willing to pay	WTP	Standardized WTP (US $ in 2021)
Bradford et al [30]	United States	HTN^b^ clinical study (1999-2000), CHF^c^ clinical study (2000-2001)	Patients were recruited from a HTN and CHF study; eligibility criteria was not stated	34	Telemedicine for HTN: peripherals send information on blood pressure, temperature, weight, heart function, and so on. Telemedicine under CHF: weight scale, blood pressure monitor, pulse oximeter, stethoscope, handheld ECG^d^, and a base PC platform	CVM^e^	At US $29.96, 32% of the population with HTN would pay out of pocket to access telemedicine. At US $29.96, >45% of the population with CHF would be willing to pay out of pocket for telemedicine access	The dollar amount when randomly varied among patients had a normal distribution with a US $20 mean (per visit)	US $29.96 per visit
Bettiga et al [33]	Italy	N/A^f,g^ (paper’s year of publication will be used as a reference for currency standardization)	Healthy patients without HTN	350	Mobile health technologies that are connected to the internet and made accessible via smartphones	Survey	N/A	N/A	N/A
Fletcher et al [36]	United Kingdom	June 3-20, 2016	Patients with self-reported HTN who were aged ≥18 years	212	Telemedicine for HTN management	DCE^h^	N/A	A total of €374.74 (US $414.76), €398.98 (US $441.59), and €673.45 (US $745.37) for a 10%, 15%, and 25% reduction in 5-year cardiovascular disease risk, respectively	US $456.99, US $486. 55, and US $821.25 for a 10%, 15%, and 25% reduction in 5-year cardiovascular disease risk, respectively
Losiouk et al [29]	Italy	A clinical trial conducted in 2015	Participants in the baseline and poststudy questionnaire were parents of children with diabetes	167	Web-based telemonitoring service that allowed parents to oversee their child	Questionnaire	N/A	Median WTP of €200 (US $265.68) annually	Median of US $246.40 annually
Park et al [37]	South Korea	Patients were surveyed from October to November 2009. Physicians were surveyed in January 2010.	Patients surveyed visited outpatient clinics at 2 tertiary care hospitals for diabetes	41	Telemedicine for diabetes management	Conjoint analysis	N/A	Marginal WTP for comprehensiveness of service is ₩16,957 (US $15.26) monthly. WTP for mobile phone over internet-based medical services is ₩15,899 (US $14.31) monthly. WTP for general hospital over physician-based services is ₩15,143 (US $13.63) monthly	US $18.43 monthly for service comprehensiveness. US $17.27 monthly for mobile phone over internet-based services. US $16.46 monthly for general hospital over physician-based services
Snoswell et al [34]	Australia	N/A^i^	Voluntary participants from the SKIN^j^ Research Project RCT^k^ were included if they owned or could access an iPhone compatible with the study’s dermoscopic attachments. Participants were excluded if in the last 5 years, they were diagnosed with melanoma	118	Direct-to-consumer teledermoscopy, which allows patients to interact directly with their dermatologists	DCE	N/A	Marginal WTP of Aus $1.18 (US $0.88) to switch from a GP^l^ visitation to mobile teledermoscopy; WTP of Aus $43 (US $32.14) to switch from a GP to a dermatologist; WTP of Aus $117 (US $87.46) to switch to an increased chance of melanoma detection	Marginal WTP of US $0.89 to switch from a GP visitation to mobile teledermoscopy; WTP of US $32.25 to switch from a GP to a dermatologist; WTP of US $87.75 to switch to an increased chance of melanoma detection
Bergmo and Wangberg [38]	Norway	The RCT was conducted from 2002 to 2003	The study’s participants were aged ≥18 years who had internet access and were keen on communicating with their GP electronically	151	Intervention groups were given access to an electronic communication system for communication with their GP	Questionnaire	Of participants, 51% expressed a positive WTP, 21% expressed a WTP of 0, and 28% declined to answer	The mean WTP for the intervention group is €4.52 (US $5.11), whereas that of the control group is €6.78 (US $7.66). WTP are expressed per web-based consultation session	The intervention group has a mean WTP of US $7.36, and the control group has a mean WTP of US $11.04
Scherrenberg et al [39]	Belgium	July to August 2020	Patients From Jessa Hospital	93	Remote cardiac rehabilitation exposure via telephone, video consultations. or live exercise	DCE	Of patients, 70% were willing to pay as much for telerehabilitation as center-based CR^m^	N/A	N/A
Ramchandran et al [31]	United States	2017	Participants had diabetes, had to be cognitively and medically fit to be interviewed or participate in the focus group held in English. Participants had to have a dilated eye examination, be assessed through teleophthalmology, or did not visit an eye physician in the past 2 years	23	Teleophthalmology, which utilizes a camera-based retinopathy exam in noneye care settings for remote image assessment	Survey	Of patients, >50% indicated their WTP to be US $32.38 or US $43.18	WTP was the amount patients usually copay (not stated)	Of patients, >50% indicated their WTP to be US $32.38 or US $43.18
Spinks et al [35]	Australia	N/A^n^	To be included, participants had to be aged 50-64 years, reside in Queensland, and have moderate or high melanoma risk	35	Teledermoscopy images for review by teledermatologists	DCE	N/A	Participants had a WTP of Aus $110 (US $101.20) to move from choosing between SSE^o^, skin cancer clinic, and GP screening to a scenario where teledermoscopy and dermatologists are offered	Participants had a WTP of US $89.70 to move from choosing between SSE, skin cancer, clinic and GP screening to a scenario where teledermoscopy and dermatologists are offered
Bradford et al [32]	United States	Clinical trial conducted from 2000 to 2001.	Patients with CHF discharged from CHF-relevant inpatient stays	126	A PC-dependent system that collected clinical data for care and monitoring of patients with CHF	DBDC^p^ CVM	Of patients, 55% had a WTP of US $29.96 for telemedicine rather than in-person care at the physician’s office. Of patients, 19% had a WTP of US $59.91 for telemedicine rather than in-person care at the physician’s office	WTP of US $20 and US $40 per visit	WTP of US $29.96 and US $59.91 per visit

^a^WTP: willingness to pay.

^b^HTN: hypertension.

^c^CHF: chronic heart failure.

^d^ECG: electrocardiogram.

^e^CVM: contingent valuation method.

^f^N/A: not applicable.

^g^The paper’s year of publication in 2020 will be cited as the year in which the study is conducted, as the time frame for when the intervention was conducted was not provided.

^h^DCE: discrete choice experiment.

^i^The paper’s year of publication in 2018 will be cited as the year in which the study was conducted, as the year of study was not reported in the paper.

^j^SKIN: Skin Innovation.

^k^RCT: randomized control trial.

^l^GP: general practitioner.

^m^CR: cardiac rehabilitation.

^n^The paper’s year of publication in 2016 will be cited as the year in which the study was conducted, as the year of study was not reported in the paper.

^o^SSE: skin self-examination.

^p^DBDC: double-bounded dichotomous choice.

#### WTP for Telemedicine

The results for the WTP for telemedicine were expressed in terms of the proportion of patients who were willing to pay for the intervention, as well as the specific WTP amounts highlighted across the 11 studies (Table 1). Regarding the proportion of patients willing to pay for telemedicine, WTP percentages across the 11 studies ranged from 19% to 70%. The study by Bradford et al [32] reported 19% of patients were willing to pay for telemedicine rather than in-person care in the physician’s office when the price was raised from US $20 to US $40. In contrast, the study by Scherrenberg et al [39] expressed that 70% of patients were willing to pay as much for telerehabilitation as center-based cardiac rehabilitation.

Regarding the WTP amount, the monetary values highlighted from all the studies were standardized to a baseline currency of US $ in 2021 for comparison between studies. This was done by converting the WTP values into US $ for the same year that the study was conducted. The converted WTP values in US $ were then converted into US $ in 2021 by considering inflation. The WTP values provided by the studies ranged from US $0.89 to US $821.25. The WTP of US $0.89 was the marginal WTP arising from switching from a general practitioner (GP) visitation to mobile teledermoscopy, whereas the US $821.25 was the annual WTP for a 25% reduction in 5-year cardiovascular disease risk. Different metrics for the WTP values were provided across the studies, for example, WTP per month, WTP per year, and WTP per session.

#### Factors Associated With the WTP

Across the studies, the association between different sociodemographic, socioeconomic, and health service variables with WTP was reported. For the purpose of this review, we have summarized variables that were reported by at least two included studies, namely, age, income, gender, travel time, marital status, and ethnicity (Table 2) The correlations between age and distance from health facilities with the WTP for telemedicine were negative (ie, the higher the age, the lower the WTP for telemedicine) and positive (ie, the longer the traveling distance, the higher the WTP for telemedicine), respectively. Both correlations were statistically significant. Whereas being female and having higher income were associated with higher WTP, being married was associated with a lower WTP for telemedicine. However, these correlations are not statistically significant. For ethnicity, although there was conflicting evidence from 2 separate studies, both of these estimates were not statistically significant.

**Table 2:** Factors associated with WTP^a^ in the included studies.

Study	Demographics					Socioeconomic income		Health service distance to preferred health facility
	Gender (female)	Age	Married	Ethnicity				
Bradford et al [30]	+^b^	−−^c^	−^d^	−	+		++^e^	
Bettiga et al [33]	N/A^f^	N/A	N/A	N/A	N/A		N/A	
Fletcher et al [36]	N/A	N/A	N/A	N/A	N/A		N/A	
Losiouk et al [29]	N/A	N/A	N/A	N/A	N/A		N/A	
Park et al [37]	N/A	−−	N/A	N/A	N/A		N/A	
Snoswell et al [34]	N/A	N/A	N/A	N/A	N/A		N/A	
Bergmo and Wangberg [38]	N/A	++	N/A	N/A	+		N/A	
Scherrenberg et al [39]	N/A	N/A	N/A	N/A	N/A		N/A	
Ramchandran et al [31]	N/A	N/A	N/A	N/A	N/A		N/A	
Spinks et al [35]	N/A	N/A	N/A	N/A	N/A		N/A	
Bradford et al [32]	+	−−	−	+	+		++	

^a^WTP: willingness to pay.

^b^The effect of the variable is positive and nonsignificant.

^c^The effect of the variable is negative and significant.

^d^The effect of the variable is negative and nonsignificant.

^e^The effect of the variable is positive and significant.

^f^N/A: not applicable; the effect of the variable is not applicable to this study.

### Quality Appraisal

The details of the quality assessment conducted for the 11 included studies are presented in Table 3. While the total number of *Yes* responses under each study’s quality assessment ranged from 4 to 10 (range 0-12), the total number of *No* responses ranged from 2 to 6 (range 0-12). The total number of *Can’t tell* responses yielded by each study’s quality assessment ranged from 0 to 3 (range 0-12). Across the 11 studies, 3 (27%) studies [34,35,37] received the highest rating of 10 *Yes* responses, whereas 2 (18%) studies [30,39] received the lowest rating of 5 *Yes* responses. All the studies except 1 [33] received a *Yes* response to the question on whether the study has a clearly focused question that helps provide relevant context to the reader in understanding the study. All the studies except 1 [35] received a *No* response to the question on whether there were prestudy considerations of statistical power. Across the board, this may not be strictly followed or widely adopted, potentially owing to the difficulty in sampling sufficient patients who met the studies’ selection criteria. All studies except 1 [39] received a *Yes* response for whether statistical significance was assessed. All studies received a *Yes* response for the applicability of results to the reviewer’s organization as findings were found to be relevant to the Singapore context. This is in consideration of the chronic disease prevalence which accounted for 29.3% of deaths in 2019, highlighting the importance of exploring the WTP for telemedicine interventions in the context of chronic diseases [40].

**Table 3:** Quality appraisal of the included studies.

Question	Bradford et al [30]	Bettiga et al [33]	Fletcher et al [36]	Losiouk et al [29]	Park et al [37]	Snoswell et al [34]	Bergmo and Wangberg [38]	Scherrenberg et al [39]	Ramchandran et al [31]	Spinks et al [35]	Bradford et al [32]
1. Did the study address a clearly focused question or issue?	Yes	No	Yes	Yes	Yes	Yes	Yes	Yes	Yes	Yes	Yes
2. Is the research method (study design) appropriate for answering the research question?	Yes	Yes	Yes	Yes	Yes	Yes	Cannot tell	Yes	Cannot tell	Yes	Yes
3. Is the method for the selection of the participants (employees, teams, divisions, and organizations) clearly described?	Cannot tell	Cannot tell	Yes	Cannot tell	Yes	Yes	Yes	Yes	Yes	Yes	Yes
4. Could the way the sample was obtained introduce (selection) bias	Cannot tell	Cannot tell	Yes	Yes	Yes	Yes	Yes	Yes	Yes	Yes	Yes
5. Was the sample of participants representative with regard to the population to which the findings will be referred?	Cannot tell	Cannot tell	Cannot tell	No	Yes	Yes	Yes	Cannot tell	Yes	Yes	Yes
6. Was the sample size based on prestudy considerations of statistical power?	No	No	No	No	No	No	No	No	No	Yes	No
7. Was a satisfactory response rate achieved?	Cannot tell	Yes	Yes	Yes	Yes	Yes	Yes	No	No	No	No
8. Are the measurements (questionnaires) likely to be valid and reliable?	Yes	Yes	Yes	No	Yes	Yes	No	No	Yes	No	Yes
9. Was the statistical significance assessed?	Yes	Yes	Yes	Yes	Yes	Yes	Yes	No	Yes	Yes	Yes
10. Are CIs given for the main results?	No	No	Yes	No	No	Yes	Yes	No	No	Yes	No
11. Could there be confounding factors that have not been accounted for?	No	Yes	No	Yes	Yes	No	Cannot tell	No	Yes	Yes	Yes
12. Can the results be applied to your organization?	Yes	Yes	Yes	Yes	Yes	Yes	Yes	Yes	Yes	Yes	Yes

## Discussion

### Principal Findings

To our knowledge, this study is the first systematic review summarizing the WTP in patients with chronic diseases for telemedicine in high-income settings, and the first systematic review summarizing the factors associated with WTP. We reported the proportion willing to pay for a telemedicine intervention to vary between 19% and 70%, with the WTP amount ranging from US $0.89 to US $821.25. In addition, we found age and distance to preferred health facility as the only reported factors to be significantly associated with the WTP for telemedicine interventions. Although gender, marital status, ethnicity, and income were reported to be associated with the WTP for telemedicine intervention, this association was not statistically significant.

For age, whereas 3 studies [30,37,38] reported a statistically significant negative correlation with the WTP for telemedicine intervention, 1 [38] reported a statistically significant positive correlation. The former is consistent with the literature, as adults aged >65 years are reported to be less inclined to use technology-based interventions than their younger counterparts [41]. This may be attributed to how pensioners tend to fear incurring high costs that come with purchasing electronics or investing in home-based health monitoring systems required by some telemedicine interventions [42]. This may also be attributed to the resistance of older patients toward new technologies, which may explain lower rates of acceptance for such technology-enabled interventions. Older patients also tend to prefer personal contact with health care providers, perceiving distance-based services such as telemedicine to be insufficient to meet this need [43], thus potentially lowering their WTP for telemedicine. On the contrary, Bergmo and Wangberg [38] reported older age to be significantly associated with a higher WTP for telemedicine services. However, on further reviewing their sample, we found that most included participants were high daily internet users, which may not be representative of the general population, including the older subgroup. Furthermore, more than half of the sample was college educated, which is reported in the literature to increase the acceptance of technology [44]. Hence, for reporting the association of age with the WTP for telemedicine, this study was identified as a potential outlier, and our study concludes a negative and statistically significant correlation following the consistency in results reported by other included studies [30,32,37]. For the variable distance to preferred health facility, 2 studies [30,32] reported a statistically significant positive correlation where further distance is associated with a higher WTP for telemedicine intervention. This WTP may be explained by the convenience afforded by telemedicine services, which may be conducted remotely in patients’ homes. Convenience was also reported as the main determinant for WTP for people aged ≥18 years looking to have consultations with their physicians over video conferencing [45]. This WTP may also be explained by how telemedicine was found to reduce associated financial burdens by reducing traveling costs, as telemedicine is conducted remotely [46], thus reducing costs to productivity [47], which patients are likely to be willing to pay to mitigate.

Moving forward with recommendations on how to enhance the WTP for telemedicine, there are also several other trends in patients’ WTP for telemedicine we could identify as we drew comparisons across the 11 studies. These pertain to the influence of prior telemedicine exposure, the impact of telemedicine costs, and the importance of patient education in contributing to WTP.

Across 3 studies [29,38,39], participants were exposed to the telemedicine intervention before being asked about their WTP for telemedicine. With the availability of this information, we can review the implications of having prior exposure on WTP. Losiouk et al [29] reported a median WTP of US $246.40 annually by parents of children with diabetes who were enrolled into a clinical trial for telemonitoring. In the second study [38], the control group communicated with their GPs as per usual (office visitations, and telephones), whereas the intervention group had access to a messaging system for a period of 1 year. Approximately 51% (77/151) had expressed a WTP with a positive value for the telemedicine intervention. In the third study [39], out of all the patients who underwent cardiac telerehabilitation sessions, approximately 70% (highest proportion across all 11 reviewed studies) were willing to pay as much for telerehabilitation as compared with a center-based session for cardiac rehabilitation. Considering that the proportion of patients willing to pay for telemedicine in this review ranged from 19% to 70%, the proportion of people who were willing to pay provided by studies with prior exposure to telemedicine intervention were relatively on the higher side. A similar trend was observed for the WTP values, which ranged from US $0.89 to US $821.25 in this review. The values provided by the study with prior exposure to telemedicine intervention were on the higher side as well. The findings from our systematic review therefore suggest that experience with the telemedicine technology in question may contribute to a greater WTP by patients. Further research may therefore explore the extent to which exposure to a telemedicine intervention may influence the WTP for telemedicine, as well as the differential impact on patients of different demographics (age, gender, etc).

Telemedicine costs were cited as a major barrier to access by 36% (4/11) of the studies. According to 1 study [37], the attribute ranked most highly in importance was cost, which had a relative importance estimated at 29%. According to another study [34], the preference weight for the telemedicine cost attribute was extremely significant at *P*=.001. According to Ramchandran et al [31], almost all participants shared that their limited insurance coverage for medical care, having fixed income and limited budget were barriers for obtaining dilated eye examinations. Many patients expressed their wish to know the cost of the examination before deciding and would be more keen on participating if they knew that insurance would pay for the service. A few stated that they would pay for eye exams because they knew the value of it if they were able to afford it. In view of the overwhelming evidence in favor of lowering telemedicine costs and prices, this spotlights the need to supply provisions that promote telemedicine access and mitigate the risks of falling into income poverty, which is no exception even for high-income countries [48]. In particular, the effect of reimbursement as a provision had been demonstrated in how a notable barrier in telehealth adoption in the United States is the shortage of significant Medicare, Medicaid, and commercial insurance reimbursements. Cost reductions in general have also been found to contribute to higher telehealth implementation [7]. In view of equity concerns, interventions that allow for cost alleviation should also be more directed to groups of people whose WTP for health services is more greatly influenced by their financial ability to pay. These groups of people who are more cost-sensitive are the low-income earners, those without university education, those who are older, and those with poor health status [49].

Patient education also plays a crucial role in eliciting patient demand, acceptance, and thus the WTP for telemedicine services. It is important to raise awareness about the efficacy of telemedicine services while simultaneously addressing patient concerns, especially because telemedicine is a relatively new model of care for many, even within high-income settings. The commonalities in findings consolidated from across the 11 articles were able to provide directions for educating patients. First, it is important to highlight the potential risk reductions brought about by the ease and regularity of telemedicine-based services (ie, telemonitoring and teledermoscopy) as compared with inpatient clinical visits, which may be more infrequent or less accessible. The WTP for risk reductions had been reported by one of the studies [36], in which participants were willing to pay for larger reductions in heart disease risk, with a WTP of US $456.99, US $486.55, and US $821.25 for a 10%, 15%, and 25% reduction in 5-year cardiovascular disease risk, respectively. Another study [34] also highlighted how consumers demonstrated the largest WTP within the study of US $87.75 to switch to telemedicine alternatives when there was an increased chance of melanoma detection. Second, it is important to address the concerns that patients may have toward telemedicine that potentially limit their use. This was evidenced by Bergmo and Wangberg [38], where 48% of patients surveyed were unwilling to pay for electronic contact with GP potentially because of there being fewer than expected benefits, a hypothetical bias, or a simple preference for face-to-face consultations. Ramchandran et al [31] also found that older participants had a strong preference for seeing an eye physician personally because of the value they place on the relationship with the health care provider, as well as the perceived thoroughness and expertise of the examination. When educating patients, emphasis should therefore be placed on the reliability and quality of teleophthalmology because web-based care, which is frequently paired with remote patient monitoring, can improve physical assessments even without the physical presence of the practitioner [50]. Furthermore, there are also studies that show a trend of higher satisfaction for physician interpersonal skills and patient-centered communications during telemedicine [51], which may be meaningful to consider. Third, the additional benefits of telemedicine that patients themselves may not immediately foresee or intuitively consider may be brought to their awareness. An example of such additional benefits that can be highlighted to patients during patient education interventions would be the reduction in parental fatigue, which is deemed the worst effect of diabetes management yet the most improvable item in daily telemonitoring when measured during the postintervention survey.

### Strengths

The following are the strengths of our review. First, to our knowledge, we are the first to conduct a systematic review on the WTP for telemedicine among patients with chronic diseases in high-income settings, thus providing a precedence for which this topic may be further explored. Second, our study used multiple databases, namely, PubMed, PsycINFO, Embase, and EconLit. This allowed for the comprehensive capture of resources. Third, as our review was conducted in accordance with the PRISMA guidelines, it is systematic and transparent. Fourth, the risk of bias was conducted for the 11 included studies following the Centre for Evidence-based Management guidelines [28]. Having 2 authors review the quality of included studies further helped to strengthen the reliability of the risk of bias assessment conducted. Finally, we did not place limits on the date of publication when it came to the selection of studies for inclusion in our review.

### Limitations

The following are our study limitations. The main limitation was the heterogeneity across the studies included in this review, which made it hard to draw conclusions beyond descriptive comparisons. First, there was no consistency in reporting the outcomes of interest, as some studies did not report the proportion of patients willing to pay for telemedicine [29,33-37], whereas some studies did not report the WTP values [33,39]. Because of this missing information, the general conclusions on WTP values or proportion of patients willing to pay for telemedicine may not necessarily be applicable to all the studies included in our systematic review. Second, with reference to Table 2, only 36% (4/11) of the studies [30,32,37,38] provided information on how specific variables correlated with the WTP for telemedicine. This limited the amount of information available to draw conclusions on factors associated with WTP. Third, the diseases represented in the selected studies may be broadly classified into diabetes, cardiovascular diseases, and skin cancer, thus only accounting for a limited number of diseases. Fourth, different methods were used in eliciting WTP across the studies, each with their own limitations as well with potential implications on the validity of the WTP values collected. Another limitation was the inclusion of articles published only in English and the exclusion of gray literature, which may have implications on the comprehensiveness of the reviewed evidence. There are also inherent differences in health care systems, financing of health care, and socioeconomic context across included studies from different countries. Overall, we acknowledge the large heterogeneity in patient populations, interventions, health care settings, interventions, and outcome measures across the 11 studies, which may potentially limit the generalizability of our findings. However, as the literature on the WTP for telemedicine is sparse at the moment, we were unable to focus on selecting papers with specific interventions, patient populations, settings, and outcome measures in this systematic review.

We also agree and acknowledge that it would be meaningful to delve more into the diverse and emerging forms of telehealth. Telemedicine technologies have developed to include a range of services such as synchronous and asynchronous consultations [52], which are of interest as they represent the latest advances in telecommunication technology, and networks in health care and promise in service delivery [53]. Artificial intelligence chatbots have also been found to provide a personalized medical consultation experience, provide immediate access to medical information, provide diagnosis recommendations, and connect patients to health care providers beyond their immediate communities [54]. However, WTP studies involving novel telemedicine technologies such as synchronous and asynchronous consultations and artificial intelligence chatbots are not yet well established, resulting in a limited range of telemedicine services represented in our paper despite the emergence of such forms of telemedicine over the years. Hence, we would recommend that further WTP research for telemedicine can compile their findings for the more novel telemedicine forms such as the ones we have identified. This would be a good starting point before empirical evidence may be synthesized in subsequent systematic reviews.

### Conclusions

On the basis of the findings of this systematic review on the WTP for telemedicine among patients with chronic health conditions, we conclude that the WTP for telemedicine varied considerably across the literature, ranging from 19% to 70%. In addition, we found age and distance to preferred health facility as the only reported factors to be significantly associated with the WTP for telemedicine interventions. The following are the practical recommendations based on our findings, which may be used to guide future interventions to boost the WTP for telemedicine. Prior exposure to the telemedicine intervention, as well as mitigating telemedicine costs, where possible, may enhance the acceptability and WTP for such interventions. Patient education was also found to be important for raising awareness on telemedicine’s benefits and addressing patients’ concerns. In view of the heterogeneity in the existing literature on WTP, future research efforts should focus on standardizing the conduct and reporting of such WTP studies so as to facilitate generalization of findings, pooling of effect estimates and generating of actionable insights to enhance the outreach of telemedicine interventions to patients with chronic diseases.

## Appendix

Multimedia Appendix 1 PRISMA (Preferred Reporting Items for Systematic Reviews and Meta-Analyses) study flowchart.

Multimedia Appendix 2 Search strategy for the PubMed database.

## Abbreviations

CVM: contingent valuation method
DCE: discrete choice experiment
GP: general practitioner
PRISMA: Preferred Reporting Items for Systematic Reviews and Meta-Analyses
WTP: willingness to pay
