# Supplementary figures and images for: The Willingness to Pay for Telemedicine Among Patients With Chronic Diseases: Systematic Review
Source: J Med Internet Res. 2022 Apr 13;24(4):e33372. doi: 10.2196/33372 (PMC9047785; doi:10.2196/33372)

**FIGURE**

**Figure 1. Prisma Study Flow Chart**


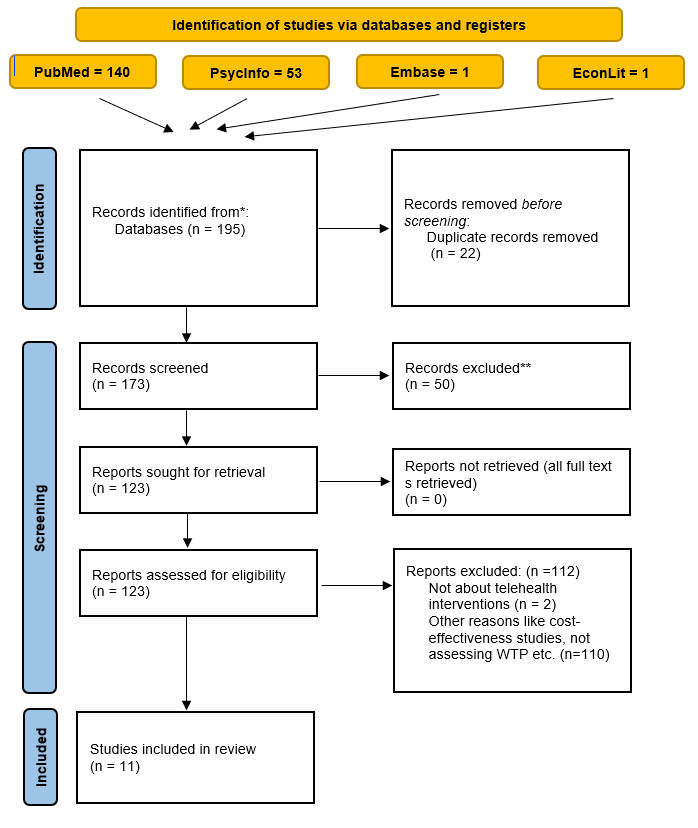

Supplement: Multimedia Appendix 1 [file jmir_v24i4e33372_app1.docx]
