# Supplementary material for: The Willingness to Pay for Telemedicine Among Patients With Chronic Diseases: Systematic Review
Source: J Med Internet Res. 2022 Apr 13;24(4):e33372. doi: 10.2196/33372 (PMC9047785; doi:10.2196/33372)
Supplement: Multimedia Appendix 2 [file jmir_v24i4e33372_app2.docx]

**APPENDIX**

**Appendix 1. Search Strategy for PubMed Database**

Key search terms:

Willingness to pay, telemedicine, chronic disease

PubMed Search Strategy

**Willingness to Pay**

"willingness to pay"[tiab] OR "willingness-to-pay"[tiab] OR "WTP"[tiab] OR "demand"[tiab] OR "contingent valuation*"[tiab] OR "cvm"[tiab] OR "discrete choice experiment"[tiab] OR "dce"[tiab]

**Telemedicine**

"telemedicine"[MeSH] OR "telemedicine"[tiab] OR "telehealth"[tiab] OR "telemonitor*"[tiab] OR "telehealthcare" OR "telecare"[tiab] OR "mobile health"[tiab] OR "telehomecare" [tiab] OR "ehealth"[tiab] OR "mobile telemedicine"[tiab] OR "telemanagement"[tiab] OR "ehealth"[tiab]

**Chronic disease**

("chronic disease*"[tiab] OR "chronic illness*"[tiab] "chronic disorder*"[tiab] OR "cardiovascular diseas*"[tiab] OR "CVD"[tiab] OR "chronic lung diseas*"[tiab] OR "diabetes mellitus"[tiab] OR "diabetes"[tiab] OR "congestive heart failure"[tiab] OR "asthma"[tiab] OR "chronic obstructive pulmonary disease*"[tiab] OR "COPD"[tiab] OR "cancer*"[tiab] OR stroke[tiab] OR "hyperlipidemia" [tiab] OR "lipid disorder*" [tiab] OR schizophrenia[tiab] OR depression[tiab] OR "bipolar disorder"[tiab] OR dementia[tiab] OR anxiety[tiab] OR "parkinson's disease"[tiab] OR "chronic kidney disease"[tiab] OR epilepsy[tiab] OR osteoporosis[tiab] OR psoriasis[tiab] OR "rheumatic arthritis"[tiab] OR "ischemic heart disease*"[tiab] OR "hyperten*" [tiab] "High blood pressure"[tiab] OR "systolic blood pressure"[tiab] OR "diastolic blood pressure"[tiab] OR "raised blood pressure"[tiab]) OR ("Asthma"[Mesh] OR "Pulmonary Disease, Chronic Obstructive"[Mesh] OR "Cardiovascular Diseases"[Mesh] OR "Diabetes Mellitus, Type 2"[Mesh] OR "Heart Failure"[Mesh] OR "Neoplasms"[Mesh] OR "Hyperlipidemias"[Mesh] OR "Schizophrenia"[Mesh] OR "Depressive Disorder, Major"[Mesh] OR "Bipolar Disorder"[Mesh] OR "Dementia"[Mesh] OR "Anxiety Disorders"[Mesh] OR "Parkinson Disease"[Mesh] OR "Renal Insufficiency, Chronic"[Mesh] OR "Kidney Failure, Chronic"[Mesh] OR "Epilepsy"[Mesh] OR "Osteoporosis"[Mesh] OR "Psoriasis"[Mesh] OR "Rheumatic Fever"[Mesh] OR "Myocardial Ischemia"[Mesh] OR "Chronic Disease"[Mesh] OR "Noncommunicable Diseases"[Mesh] OR "Hypertension"[Mesh])
